# Supplementary material for: Did the evidence-based intervention (EBI) programme reduce inappropriate procedures, lessen unwarranted variation or lead to spill-over effects in the National Health Service?
Source: PLoS One. 2023 Sep 1;18(9):e0290996. doi: 10.1371/journal.pone.0290996 (PMC10473535; doi:10.1371/journal.pone.0290996)
Supplement: S1 File — (DOCX) [file pone.0290996.s001.docx]

# Supplementary material 1

## SCV equation

**SCV =**$\left\{ \frac{\boldsymbol{\Sigma}_{\boldsymbol{i}} \frac{\left( \boldsymbol{O}_{\boldsymbol{i}}\boldsymbol{-}\boldsymbol{E}_{\boldsymbol{i}} \right)^{\boldsymbol{2}}}{\boldsymbol{E}_{\boldsymbol{i}}^{\boldsymbol{2}}}\boldsymbol{-}\boldsymbol{\Sigma}_{\boldsymbol{i}}\frac{\boldsymbol{1}}{\boldsymbol{E}_{\boldsymbol{i}}}}{\boldsymbol{n-1}} \right\}\boldsymbol{\times100}$

Previous studies applying SCV scores have no consensus on the scaling nor the threshold to classify the magnitude of SCV scores [1, 2]. SCV in this work was calculated using the formula from Freibel et al. (2018) [1] Our focus is analysing changes in SCV scores and therefore the magnitude of variation is of less importance, however we will use SCV thresholds of >5 as high variation and >10 as very high variation to categorise scores [1, 2].

## 10 Year data plots

Figure 1 Snoring Surgery

Figure 2 Heavy Menstrual Bleeding

Figure 3 Knee Arthroscopy

Figure 4 Injections for lower back pain (Denervation – substitute procedure)

Figure 5 Breast Reduction

Figure 6 Benign Skin Lesions

Figure 7 Grommets

Figure 8 Tonsillectomy

Figure 9 Haemorrhoid Surgery

Figure 10 Hysterectomy

Figure 11 Chalazion Removal

Figure 12 Subacromial shoulder decompression


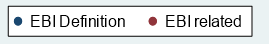


Figure 13 Carpel Tunnel


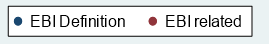


Figure 14 Dupuytrens Contracture


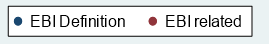


Figure 15 Ganglion Excision


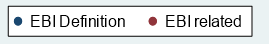

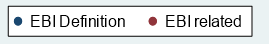

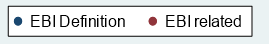


Figure 16 Trigger Finger Release

Figure 17 Varicose Veins

## HES Codes for extracting procedure episodes

**A. Snoring Surgery in the Absence of Sleep Apnoea**

| *Field* | *Code* | *Description* |
| --- | --- | --- |
| *[[* |  |  |
| *(((* |  |  |
| Primary Procedure | F324 | Operations on uvula NEC |
| OR |  |  |
| Primary Procedure | F325 | Uvulopalatopharyngoplasty |
| OR |  |  |
| Primary Procedure | F326 | Uvulopalatoplasty |
| ))) |  |  |
| OR |  |  |
| ((( |  |  |
| Primary Procedure | F328 | Other specified other operations on palate |
| AND |  |  |
| Primary Diagnosis | R065 | Mouth breathing |
| ))) |  |  |
| ]] |  |  |
| AND |  |  |
| NOT Primary Diagnosis | G473 | NOT sleep apnea |
| AND |  |  |
| Adult Age | 19-120 |  |

**B. D&C for Heavy Menstrual Bleeding**

| *Field* | *Code* | *Description* |
| --- | --- | --- |
| Primary Procedure | Q103 | Dilation of cervix uteri and curettage of uterus NEC |
| AND |  |  |
| NOT Any Diagnosis | O0[0-8] | NOT difficulties in pregnancy |
| AND |  |  |
| NOT Any Diagnosis | O6[0-9] | NOT difficulties in delivery |
| AND |  |  |
| NOT Any Diagnosis | O7[0-5] | NOT difficulties during labour |

**C. Knee Arthroscopy for Osteoarthritis**

| *Field* | *Code* | *Description* |
| --- | --- | --- |
| ((( |  |  |
| Primary Procedure | W82[12389] | Therapeutic endoscopic operations on semilunar cartilage |
| OR |  |  |
| Primary Procedure | W85[12389] | Therapeutic endoscopic operations on cavity of knee joint |
| OR |  |  |
| Primary Procedure | W879 | Unspecified diagnostic endoscopic examination of knee joint |
| ))) |  |  |
| OR |  |  |
| [[ |  |  |
| ((( |  |  |
| Primary Procedure | W83 | Therapeutic endoscopic operations on other articular cartilage |
| OR |  |  |
| Primary Procedure | W84[1234] | Endoscopic operation on intra-articular cartilage or synovial plica |
| OR |  |  |
| Primary Procedure | W861 | Endoscopic removal of loose body from joint NEC |
| ))) |  |  |
| AND |  |  |
| ((( |  |  |
| Any Procedure | Z846 | Knee Joint |
| OR |  |  |
| Any Procedure | O132 | Knee NEC |
| ))) |  |  |
| ]] |  |  |
| OR |  |  |
| [[ |  |  |
| Primary Procedure | W901 | Aspiration of joint |
| AND |  |  |
| ((( |  |  |
| Any Procedure | O132 | Knee NEC |
| OR |  |  |
| Any Procedure | Z12[123] |  |
| OR |  |  |
| Any Procedure | Z504 | Skin of leg NEC |
| OR |  |  |
| Any Procedure | Z577 | Hamstring |
| OR |  |  |
| Any Procedure | Z58 | Muscle of lower leg |
| OR |  |  |
| Any Procedure | Z77[12489] | Tibia |
| OR |  |  |
| Any Procedure | Z78[1236789] | Bone of lower leg |
| OR |  |  |
| Any Procedure | Z84[456] | Patellofemoral, tibiofemoral, or knee joint |
| Or |  |  |
| Any Procedure | Z851 | Upper tibiofibular joint |
| Or |  |  |
| Any Procedure | Z904 | Lower leg NEC |
| ))) |  |  |
| ]] |  |  |
| AND |  |  |
| Primary Diagnosis | M1[57] | Arthrosis or gonarthrosis |
| AND |  |  |
| Adult Age |  | 19-120 |

**D. Injection for Non-specific Low Back Pain**

| *Field* | *Code* | *Description* |
| --- | --- | --- |
| *(((* |  |  |
| Primary Procedure | A52[1289] | Therapeutic lumbar, sacral, other, or unspecified epidural injection |
| OR |  |  |
| Primary Procedure | A577 | Injection of therapeutic substance around spinal nerve root |
| OR |  |  |
| Primary Procedure | A735 | Injection of therapeutic substance around peripheral nerve |
| OR |  |  |
| Primary Procedure | V544 | Injection around spinal facet of spine |
| ))) |  |  |
| AND |  |  |
| ((( |  |  |
| Primary Diagnosis | M51[89] | Specified or unspecified intervertebral disc disorder |
| OR |  |  |
| Primary Diagnosis | M54[59] | Low back pain and unspecified dorsalgia |
| ))) |  |  |
| AND |  |  |
| ((( |  |  |
| Any Procedure | Z76[567] | Lumbar vertebra, lumbosacral joint, or sacrococcygeal joint |
| OR |  |  |
| Any Procedure | Z993 | Intervertebral disc of lumbar spine |
| ))) |  |  |

**E. Breast Reduction Surgery**

| *Field* | *Code* | *Description* |
| --- | --- | --- |
| Primary Procedure | B311 | Breast reduction |
| AND |  |  |
| NOT Any Diagnosis | Z853 | Personal history of cancer |

**F. Removal of Benign Skin Lesions**

| *Field* | *Code* | *Description* |
| --- | --- | --- |
| ((( |  |  |
| Primary Procedure | S06[3-9] | Excision of lesion of skin |
| OR |  |  |
| Primary Procedure | S08[12389] | Curretage of lesion of skin |
| OR |  |  |
| Primary Procedure | S09[1234589] | Laser/infrared/photodestruction of lesion of skin |
| OR |  |  |
| Primary Procedure | S10[12] | Cauterisation or cryotherapy of lesion of skin of head or neck |
| OR |  |  |
| Primary Procedure | S11[12] | Cauterisation or cryotherapy of lesion of skin |
| OR |  |  |
| Primary Procedure | D02[1289] | Excision, destruction, extirpation of external ear |
| ))) |  |  |
| AND |  |  |
| NOT Any Diagnosis | Z858 | Z858 Personal history of malignant neoplasms |

**G. Grommets for Glue Ear in Children**

| *Field* | *Code* | *Description* |
| --- | --- | --- |
| ((( |  |  |
| Primary Procedure | D151 | Myringotomy with insertion of ventilation tube through tympanic membrane |
| OR |  |  |
| Primary Procedure | D289 | Unspecified other operations on ear |
| ))) |  |  |
| AND |  |  |
| ((( |  |  |
| Primary Diagnosis | H65[23] | Chronic serous or mucoid otitis media |
| OR |  |  |
| Primary Diagnosis | H66[1-9] | Chronis tubotympanic, atticoantral, or other suppurative otitis media |
| ))) |  |  |
| AND |  |  |
| ((( |  |  |
| Age | 0-18 | Children (by their definition) |
| OR |  |  |
| Age | 7001-7007 | Babies |
| ))) |  |  |

**H. Tonsillectomy for Recurrent Tonsillitis**

| *Field* | *Code* | *Description* |
| --- | --- | --- |
| ((( |  |  |
| Primary Procedure | F34[1-9] | Different types of tonsillectomy |
| OR |  |  |
| Primary Procedure | F361 | Destruction of tonsil |
| ))) |  |  |
| AND |  |  |
| NOT Any Diagnosis | G47 | Sleep disorders |
| AND |  |  |
| NOT Any Diagnosis | J36 | Peritonsillar abscess |

**I. Haemorrhoid Surgery**

| *Field* | *Code* | *Description* |
| --- | --- | --- |
| Primary Procedure | H51[12389] | Different types of excision of haemorrhoid |

**J. Hysterectomy for Heavy Menstrual Bleeding**

| *Field* | *Code* | *Description* |
| --- | --- | --- |
| ((( |  |  |
| Primary Procedure | Q07[2489] | Abdominal hysterectomy |
| OR |  |  |
| Primary Procedure | Q08[289] | Vaginal hysterectomy |
| ))) |  |  |
| AND |  |  |
| NOT Any Diagnosis | O0[0-8] | Difficulties in pregnancy |
| AND |  |  |
| NOT Any Diagnosis | O6[0-9] | Difficulties in delivery |
| AND |  |  |
| NOT Any Diagnosis | O7[0-5] | Difficulties during labour |
| AND |  |  |
| NOT Any Diagnosis | N81 | Female genital prolapse |

**K. Chalazia Removal**

| *Field* | *Code* | *Description* |
| --- | --- | --- |
| ((( |  |  |
| Primary Procedure | C12[124] | Excision, cauterisation, or curettage of lesion of eyelid |
| OR |  |  |
| Primary Procedure | C19[18] | Drainage or other specified incision of eyelid |
| ))) |  |  |
| AND |  |  |
| Primary Diagnosis | H001 | Chalazion |

**L. Decompression for shoulder pain**

| *Field* | *Code* | *Description* |
| --- | --- | --- |
| Primary Procedure | O291 | Subacromial decompression |
| AND |  |  |
| Any Procedure | Y767 | Arthroscopic approach to joint |
| AND |  |  |
| ((( |  |  |
| Primary Diagnosis | M754 | Impingement syndrome of shoulder |
| OR |  |  |
| Primary Diagnosis | M255 | Bursitis of shoulder |
| ))) |  |  |

**M. Carpal Tunnel Syndrome Release**

| *Field* | *Code* | *Description* |
| --- | --- | --- |
| Primary Procedure | A65[19] | Carpal tunnel release or unspecified release of nerve entrapment at wrist |
| AND |  |  |
| Primary Diagnosis | G560 | Carpal tunnel syndrome |

**N. Dupuytrens Contracture Release**

| *Field* | *Code* | *Description* |
| --- | --- | --- |
| ((( |  |  |
| Primary Procedure | T52[1256] | Palmar fascietomy or digital fascietomy |
| OR |  |  |
| Primary Procedure | T5[46]1 | Division of palmar fascia or dermofascietomy |
| ))) |  |  |
| AND |  |  |
| Primary Diagnosis | M720 | Palmar fascial fibromatosis [Dupuytren] |
| AND |  |  |
| Adult Age | 19-120 |  |

**O. Ganglion Excision**

| *Field* | *Code* | *Description* |
| --- | --- | --- |
| ((( |  |  |
| Primary Procedure | T59[1289] | Excision of ganglion |
| OR |  |  |
| Primary Procedure | T60[1289] | Re-excision of ganglion |
| ))) |  |  |
| AND |  |  |
| Primary Diagnosis | M674 | Ganglion |

**P. Trigger Finger Release**

| *Field* | *Code* | *Description* |
| --- | --- | --- |
| *[[[* |  |  |
| ((( |  |  |
| Primary Procedure | T69[1289] | Tenolysis or freeing of tendon |
| OR |  |  |
| Primary Procedure | T70[12] | Tenotomy |
| OR |  |  |
| Primary Procedure | T71[189] | Tenesynovectomy or excision of sheath around tendon |
| OR |  |  |
| Primary Procedure | T72[389] | Other operations on sheath of tendon |
| ))) |  |  |
| AND |  |  |
| Any Procedure | Z89[4567] | Hand, thumb, finger, multiple digits of hand |
| ]]] |  |  |
| AND |  |  |
| Primary Diagnosis | M653 | Trigger finger |
| AND |  |  |
| Adult Age | 19-120 |  |

**Q. Varicose Vein Interventions**

| *Field* | *Code* | *Description* |
| --- | --- | --- |
| ((( |  |  |
| Primary Procedure | L83[289] | Subfascial ligation or other operations for venous insufficiency |
| OR |  |  |
| Primary Procedure | L84[12345689] | Combined operations on saphenous vein |
| OR |  |  |
| Primary Procedure | L85[12389] | Ligation of saphenous/varicose vein |
| OR |  |  |
| Primary Procedure | L86[12389] | Injection/scleropothy of varicose vein |
| OR |  |  |
| Primary Procedure | L87[1-9] | Stripping, avulsion, excision, incision of varicose vein |
| OR |  |  |
| Primary Procedure | L88[12389] | Ablation or other transluminal operations on varicose vein |
| ))) |  |  |
| AND |  |  |
| Primary Diagnosis | I8[03] | Plebetitis, thrombophlebitis, or varicose veins of lower extremities |

## Identified surgical substitute procedures

**D. Injection for Non-specific Low Back Pain**

V48.5: Radiofrequency controlled thermal denervation of spinal facet joint of lumbar vertebra [RFD]

V48.6: Denervation of spinal facet joint of lumbar vertebra NEC [Denervex]

**G. Grommets for Glue Ear in Children**

D15.2, D15.3. *Myringotomy without insertion of grommet*

**H. Tonsillectomy for Recurrent Tonsillitis**

Biopsy of tonsil.F36.[2,6,8} E25.2

**I. Haemorrhoid Surgery**

More invasive procedures to destroy haemorrhoids

H52.[1, 2, 3, 4, 8, 9]

H53. [3, 8, 9]

**J. Hysterectomy for Heavy Menstrual Bleeding**

Hysteroscopy, Biopsy, Hormone Coil Insertion, Endometrial Ablation, Myomectomy

Q09.[2,3,4]

Q12.1

Q16.[2,3,4,5,6,7]

Q17.[6,7]

Q18.[1,8,9]

Q20.[2]

References

1. Friebel, R., et al., *National trends in emergency readmission rates: a longitudinal analysis of administrative data for England between 2006 and 2016.* BMJ Open, 2018. **8**(3): p. e020325.

2. Appleby, J., et al. *Variations in health care: the good, the bad and the inexplicable*. 2011; Available from: <https://www.kingsfund.org.uk/sites/default/files/field/field_publication_file/Variations-in-health-care-good-bad-inexplicable-report-The-Kings-Fund-April-2011.pdf>.
