# Supplementary material for: Did the evidence-based intervention (EBI) programme reduce inappropriate procedures, lessen unwarranted variation or lead to spill-over effects in the National Health Service?
Source: PLoS One. 2023 Sep 1;18(9):e0290996. doi: 10.1371/journal.pone.0290996 (PMC10473535; doi:10.1371/journal.pone.0290996)
Supplement: Attachment — Submitted filename: Response to Reviewers.docx [file pone.0290996.s003.docx]

Dear Editors and Reviewers,

Thank you for the opportunity to respond to the very helpful academic editor and reviewers’ comments. The comments required no significant changes or additions to our analyses or findings. Therefore, we have made clarifications, added further information, and re-worded sections of the manuscript as advised by the reviewers. See our full responses below.

We have presented our responses in the table below. The reviewers’ comments are in column one, our responses in column two and the location and full extract of the changes to the manuscript in column three.

Yours,

Joel Glynn on behalf of the authorship team.

|  | **Comment** | **Response** | **Edit** |  |
| --- | --- | --- | --- | --- |
|  | **Reviewer 1** | | |  |
|  | *Given that the previous analysis has been done by an entirely different group, I think a more explicit comparison (and a clearer sense of the differences in methods between the two papers) would also be helpful for readers. I have not read the other paper but it appears it used a ‘differences in differences’ model as opposed to the ITS design here, and it was not clear to me why the methods varied (and I assume the former design would be stronger, all other things being equal?). That might be explained more clearly in the current paper given the overlap.* | Response: We agree that more detail is needed and have added it to the paper.  We believe that both approaches have merits and that difference in differences is not necessarily the stronger approach. For example, our ITS analysis allowed us to explore changing trends over time whilst the Anderson et al. difference-in-differences approach explored overall differences between the before/after time periods. Additionally, using different methods of analysis and coming to similar conclusions strengthens the inference drawn from both papers.  Difference in differences requires a comparator. While an appropriate comparator would strengthen the analyses it is not obvious whether an appropriate comparator exists in this instance. The Anderson. et.al paper used the EBI programme’s list two interventions as similarly low-value treatments not targeted by the EBI at the time of analyses. However, there are differences between interventions included in list one and list two, for example, list one captured procedures that were widely agreed upon as being of ‘low value’ and were already on a downward trajectory, whereas list two included procedures that were not already reducing. Therefore, lacking a clear comparator group, we decided that ITS would be an appropriate approach. | **Lines 337 to 345**  We used an ITS analysis in contrast to the difference in differences method used by Anderson et al. (16). Difference in differences requires a comparison group of similar procedures not affected by EBI. Anderson et al. use EBI list two interventions as a comparison group not targeted by EBI at the time of analysis. However, there are important differences between the list one and list two procedures. List two included procedures that were not already reducing and less widely agreed upon as ‘low value’ compared to list one. Therefore, in the absence of a clear comparator, we decide the ITS would be an appropriate approach and the assumption of a continuing trend is plausible. |  |
|  | *I think a short description (maybe in a Box) of the procedures and clinical criteria could be provided – not everyone will have the capacity to seek out additional references and I would prefer that the paper is self-contained.* | We agree with the reviewer that the paper should be as self-contained as possible. The full list of procedures is included in Table 1. We have added a footnote to Table 1 containing the NHS England citation and URL where readers can view the full procedure descriptions and criteria which are publicly available. The procedure descriptions and clinical criteria are very lengthy and cannot be simply summarised in a box. | **Table 1, page 12.** |  |
|  | *I would also like a little more on the mechanisms of the EBI – the Discussion suggests it involved ‘publishing nationwide guidance’ but that sounded quite passive? Is it plausible that the methods used would have the sort of impacts that they were looking for, especially in the context of overall reductions over time?* | We have added more detail to the manuscript.  The EBI team proposed changes to reimbursement tariffs for Category 1 (effectively not offering reimbursement except through the individual funding request mechanism), set individual targets for CCGs to meet, and proposed that prior approval be implemented for Category 2 procedures. However, with the exception of tariff changes, they were mostly passive. | **Lines 106 to 111:**  Alongside the publication of list one as statutory guidance, the EBI programme also set individual reduction targets for each CCG. Monitoring of progress towards these targets was made available through an online dashboard. The programme also recommended that reimbursement tariff payments be reduced to zero for category one procedures and that all category two procedures required prior approval by their CCG. |  |
|  | *There is a useful new framework for ‘spill over’ (see Francetic et al in Implementation Science Communications -* [*https://implementationsciencecomms.biomedcentral.com/articles/10.1186/s43058-022-00280-8*](https://implementationsciencecomms.biomedcentral.com/articles/10.1186/s43058-022-00280-8)*). I am from that group so something of a competing interest, but I wondered if the current paper might benefit linking the work to that framework if it added value.* | Thanks for drawing our attention to this framework. We agree that it is useful to place our work in the context of this framework and we have cited the framework. | **Lines 160 and 166**  Related procedures were defined as an episode of care that had the targeted EBI primary procedure code(s) but with a non-EBI diagnosis (i.e. a type of within unit spill-over in the INTENTS framework(20)).  Substitute procedures were defined as different primary procedures which could be used as an alternative to the EBI procedure to treat the same patient group (i.e. a type of between unit spill-over (20)). |  |
|  | *I am not a statistician so am not able to judge the technical quality of the analyses but they seemed rigorous and I found them clearly described.* | Thank you |  |  |
|  | *The description of the numbers of procedures on page 10 would benefit from percentages and I would link those frequencies to Table 1 so that readers could judge the volume alongside the data on changes.* | Yes, we agree percentages have been added.  Frequencies for each procedure are presented in the trend graphs in the supplementary materials. Providing frequencies for all 17 procedures was considered too lengthy to be added to the main manuscript. | **Line 232 and line 233**  Aggregating across the 17 EBI procedures, there were 447,227 procedures in 2017/18, 433,159 in 2018/19 (3.2% reduction) and 403,739 in 2019/20 (6.8% reduction, including March 2020). |  |
|  | *Is power an issue for any of these analyses? I assume for high volumes it is not, but what about lower volumes and subgroups by CCG? Are any of the analyses insufficiently precise because of numbers, or are the lower N still enough for confidence to detect policy relevant changes? Is there a sense of what size a policy related change would be to anchor the analyses?* | We apologise for this not being clear. We undertake the ITS analyses by procedure rather than by CCG. Our CCG level analysis is for the study of geographical variation only. In the analysis of geographic variation, we pool procedures (into category one or category two procedures) for this very reason. In the ITS analysis the procedure with the smallest yearly frequency is snoring surgery, in which there were approximately 630 procedures per year over our study period. Confidence intervals are provided in table 1 and act as an indication of the precision of our estimates. | No change to the manuscript. |  |
|  | *I did struggle with the suggestion in the discussion that the EBI may have had a role in continuing downward trends. How is that plausible given the data? Additionally, is the ITS even capable of assessing such an effect – the section on the ITS on page 17 suggests that this is not the case anyway? I felt that was a ‘reach’ and would prefer to see that removed unless they can justify it more clearly.* | We agree and have removed this from the discussion section. | **Removed from line 316 and 317** |  |
|  | *In the discussion of the ITS on page 17, I am not sure why the lack of control group is moderated by analyses over 17 procedures. If the design is flawed then multiple uses of the same design is not going to provide any protection (see Marion Campbell’s ‘red sock in a white wash’ analogy for bias). Maybe I am missing the logic but this did not seem plausible to me. I am happy with the ITS though (although interested in why Anderson used ‘differences in differences’).* | We don’t think the analogy is quite appropriate here, but we appreciate the point the reviewer is making. We have tried to clarify the logic in the text inserted into the manuscript. | **Lines 380 to 389**  “ITS is susceptible to bias due to unmeasured external shocks, many of these will be specific to one procedure (e.g. publication of a paper supporting or questioning efficacy). If it occurs, the bias could either inflate or deflate the apparent effect of EBI. If EBI were generally effective, and yet (as we have observed) procedure rate trends did not change post-EBI, that would imply one general external shock post-EBI that has counteracted the effect of EBI or seventeen specific external shocks have had the same counterbalancing effect for each procedure. Whilst we cannot exclude these possibilities, a simpler explanation and one that is in line with previous research (Anderson et al) using different methods, is that the EBI programme did not achieve its objectives to reduce unnecessary healthcare or geographical disparities in access to care.” |  |
|  | *There is an interesting conclusion that ‘de-adoption programmes are essential’. I understand the sentiment, although one interpretation is that wider, non-programmatic changes are leading to reductions, whereas a specific programme to drive change added nothing. I wonder if the conclusion needed changing to better reflect the actual results that they present?* | We agree with this comment and have changed the wording to reflect and say that reductions in inappropriate care are essential, rather than a specific method of reducing the use of these procedures. | **Lines 471 to 473**  De-adoption of low-value care is essential in maintaining health systems across the world. We need to better understand the enablers and barriers to effective de-adoption to support future de-adoption endeavours. |  |
|  | **Reviewer 2** | | |  |
|  | *Line 85: NHS England and Improvement -> a bit confusing as it stands for readers outside the UK (two separate entities? NHS England and NHS Improvement?)* | We have removed “and improvement” | **Line 84** |  |
|  | *Line 94: 17 surgical procedures were initially identified -> how? And how were the EBI recommendations reached?* | We have added more information about how the EBI programme settled on the 17 surgical procedures included in list one.  There is limited information available to us about the specifics of this process. Again, through qualitative interviews with the EBI programme team (work to be published) we are exploring these processes more. We have also referenced the EBI documentation where all available information on the development of the EBI programmes list one guidelines are published. | **Lines 98 to 103**  The list of 17 procedures were selected in an iterative process from a much larger list collated by 'expert working groups’ using clinical evidence from bodies such as NICE, the ‘Choosing Wisely’ programme, academic studies and local CCG work. The procedures are presented in table 1, and full details of the procedures included and clinical criteria, including the selection process, have been published in detail elsewhere (13). |  |
|  | *Line 101: Substantial -> would be good to have a clearer understanding of extent (e.g. in %)* | Thanks, we have added. | **Line 97**  …reduce procedures by 65% a total reduction of 128,000 procedures per year. |  |
|  | *Lines 106-107: how is the first task different from what Anderson et al. had already done?* | Sorry for not making this clear. The Anderson et al. paper produced a single analysis of all procedures pooled together. In our work, we analysed each procedure individually to see if there was evidence of successful de-adoption in any individual procedure. | **Line 123**  …1.) evaluating whether the EBI programme successfully promoted de-adoption in any of the 17 individual surgical procedures by analysing procedures individually |  |
|  | *Line 118: up to 24 procedures per person/case?* | This refers to ‘per episode of care’. We have added clarity. | **Lines 134 to 139**  HES presents data as episodes of care referring to periods of care under a single consultant. HES-APC records up to 24 procedures per episode captured as Office of Population Censuses and Surveys (OPCS-4) codes and up to 20 diagnoses defined by International Classification of Disease (ICD-10) codes which we used to identify eligible episodes of care (17, 18). |  |
|  | *Line 119: how you identified eligible episodes of care is not fully clear, consider elaborating with 1-2 lines* | Sorry, this was not clear at this point in the text. This process is explained in the ‘Eligible Episodes’ section further down in the paper. See the section starting on Line 150 | No Changes to the Manuscript |  |
|  | *Line 120: consider defining episode of care for this article. Is the data linked/linkable across datasets/settings on an individual patient basis? Please describe the data and how you used it for your research questions a bit more.* | Again, we apologise for the lack of clarity. | **Line 134**  HES presents data as patient episodes of care referring to periods of care under a single consultant. Therefore an individual patient may have more than one episode of care within the dataset we analyse. |  |
|  | *Line 139 onwards: consider providing an example here as well as you do below for the substitute procedures. For readers not versed in EBI, it is not necessarily intuitive why an EBI procedure code with a non-EBI diagnosis is a related procedure.* | We agree that the ‘related procedures’ have a less intuitive interpretation.  We have added an example in relation to knee arthroscopy. | **Line154 to 158**  For example, the EBI procedure knee arthroscopy relates only to the treatment of osteoarthritis, this procedure is also commonly undertaken for ‘internal derangement of the knee, these procedures are not targeted by EBI and therefore captured as related procedures in our analyses. |  |
|  | *Line 145: met with 11 surgeons -> when, selected how, how did you collect information from them etc. (see above about describing data and method. approach a bit more)* | We gathered a list of specialist surgeons that undertake the surgeries or work in the clinical areas relating to each of the EBI procedures. These were existing contacts made by members of our wider research team (that includes clinicians). We had a videocall with each to discuss the procedures exploring clinical codes and if any substitute procedures could/would likely be affected if the EBI treatment was restricted. | **Lines 160 to 164.**  To identify potential substitute procedures, using existing contacts we met with 11 specialist surgeons with expertise in one or more of the 17 EBI procedures. EBI procedures, related procedures, and substitute procedure episodes were mutually exclusive categories. |  |
|  | *Line 149: how did you set the 10% threshold (rationale)?* | This figure was chosen to avoid analysing small numbers of procedures that may occur in outpatients. We only wanted to analyse those with a significant proportion completed in the outpatient setting. | **Line 170 to 173**  However, appropriate or measurable substitute procedures were not identified for all EBI procedures, and related procedures were only analysed if they constituted a significant (>10%) proportion of the total number of the related EBI procedure performed |  |
|  | *Line 161: write out ONS, potentially explain its function* | We have edited to spell out the acronym. | **Line 185**  The model was offset using population estimates, provided by the Office for National Statistics (ONS), to control for changes in population size over the study period (21). |  |
|  | *Line 169-170: COVID-19 impact on procedures -> would it make sense to discuss a bit more in the discussion if these reductions in case numbers mean reduction of low-value procedures or increased unmet need?* | Sorry for the confusion. The study period for our analyses stops before the first COVID lockdown on the 23^rd^ March 2020 (England). Therefore, we feel it is not appropriate to discuss the COVID impacts in this piece of work. However, in future work, we are analysing EBI’s second list of procedures, and will discuss in detail and account for the impact of COVID. | No change to the manuscript. |  |
|  | *Line 172: analysis instead of analyses* | Thanks, we have made the edit. | **Line 196**  We used an interrupted time series analysis utilising segmented Poisson regression models to compare trends in pre- and post-EBI procedure rates. |  |
|  | *Line 197: Please check if there is a word missing... if "EBI list one" is a thing, perhaps good to explain what.* | Thanks, we have edited it to avoid confusion | **Line 221**  We assessed the impact of the EBI programme’s list one consultation period (July 2018- April 2019). |  |
|  | *Line 207: might it make sense to comment on the likely full year value for 2019/2020?* | We agree this could cause some confusion. Therefore, we have decided to include the full year figures available in the dataset, i.e. including March 2020 (COVID restriction effecting procedures from 23^rd^ March). Our formal ITS and variation analyses still excludes March 2020. | **Line 231 to 233**  Aggregating across the 17 EBI procedures, there were 447,227 procedures in 2017/18, 433,159 in 2018/19 (3.2% reduction) and 403,739 in 2019/20 (6.8% reduction, including March 2020). |  |
|  | *Line 210: good that you provide the range, any chance of getting a sense of distribution too without going to the Annex?* | We agree this would be helpful. We have added the median procedure as well as the range to give an idea of the distribution without listing all 17 procedures and their frequencies. | **Line 235 to 238**  The least frequent procedure was snoring surgery with 1,836 procedures over the 3 years, and the median procedure was grommets for glue ear in children with 31,020 procedures over the three years. |  |
|  | *Lines 222 onwards: consider rephrasing this period, it is a bit difficult to read.* | We have rephased to reduce the length and add clarity. | **Line 250 to 256**  Across all category one (not to be routinely commissioned) and category two procedures (commissioned only when specific criteria are met), there were 43 and 327 procedures per 100,000 in 2017/18 respectively, falling to 32 and 298 per 100,000 in 2018/19 and 23 and 259 per 100,000 in the 11 months after EBI (excision of benign skin lesions are excluded given very large numbers). |  |
|  | *Lines 239-240: would be useful to comment on why same direction and not significant are equated here* | We apologise for the confusion and have rephrased for clarity. These were not intended to be equated, we meant that some substitute/related procedures showed non-significant results, but the point estimates were in the same direction as the point estimates for the EBI procedure. | **Line 271 and 273**  In general, trend changes in these procedures were not significant and had point estimates in the same direction as the point estimates for the associated EBI procedure |  |
|  | *Line 292: move the reference bracket to line 291 after "al."* | Change made | **Line 324** |  |
|  | *Line 311-316: for readers not familiar with the EBI, it would be good to understand better what if any implementation tools accompanied the EBI and comment on potential alternatives among the ones you describe here. it would be important to reflect on this as the best possible evidence-based recommendation may not affect outcomes if not actively disseminated, and even then other barriers play a role. I think the paper would benefit from a bit more input from the literature on these issues in the discussion.* | Thank you, this would be a helpful addition and is similar to a comment posed by reviewer one.  We have added detail about the mechanisms in the introduction. As part of a larger research project, we are working to generate recommendation for de-adoption effort. We will expand on the alternatives in future work once we have undertaken our full mixed methods analysis and triangulated results with patient, clinical and commissioner input. | **Lines 106 to 111:**  Alongside the publication of list one as statutory guidance, the EBI programme also set individual reduction targets for each CCG. Monitoring of progress towards these targets was made available through an online dashboard. The programme also recommended that reimbursement tariff payments be reduced to zero for category one procedures and that all category two procedures required prior approval by their CCG. |  |
|  | *Lines 328-330: does this mean you actually had a national sample? perhaps good to add on representativity of data in the methods* | Yes, HES APC data capture all episodes of NHS-funded health care in an English hospital. Therefore, it is fully representative of English patients. This is highlighted in the strengths/limitations section on page 18 lines 346-352. | No change to the manuscript. |  |
|  | *Lines 352-354: This needs to be moved to methods and get a bit more detail.* | Thanks for this comment, it is a definite weakness with the outpatient dataset, however this only affects a few procedures. We mention in the methods (lines 138 to 134) that primary diagnosis is very poorly captured in the HES outpatient dataset, including assumptions as to whether these should be included as EBI interventions in our analyses. | No change to the manuscript. |  |
|  | *Line 359: analysed instead of identified?* | Thanks, we have edited. | **Line 408**  The majority of procedures included in EBI’s list one guidelines were already on a downward trajectory prior to their publication |  |
|  | *Line 360-362: polish style a bit (now procedures comes up twice in short interval)* | We have replaced this section of text based on an earlier comment. | **Line 380 to 389**  ITS is susceptible to bias due to unmeasured external shocks, many of these will be specific to one procedure (e.g. publication of a paper supporting or questioning efficacy). If it occurs, the bias could either inflate or deflate the apparent effect of EBI. If EBI were generally effective, and yet (as we have observed) procedure rate trends did not change post-EBI, that would imply either one general external shock post-EBI that has counteracted the effect of EBI or seventeen specific external shocks have had the same counterbalancing effect for each procedure. Whilst we cannot exclude these possibilities, a simpler explanation and one that is in line with previous research [16] using different methods, is that the EBI programme did not achieve its objectives to reduce unnecessary healthcare or geographical disparities in access to care. |  |
|  | *Line 366 onwards: should that not be coupled with monitoring efforts to recognise if deimplementation levels are/become satisfactory, though? From what point onwards is a downward trend considered sufficient evidence of a process of deadoption?* | This is a helpful point that we have added to the text. | **Line 418**  A simple monitoring of rates may be more appropriate in these cases. |  |
|  | *Line 372: well-developed de-adoption ->this is a valuation for which you (rightly) do not provide evidence here. Consider rigorously developed, or successful, as alternatives.* | We have replaced with rigorously | **Line 423**  Previous rigorously developed de-adoption initiatives, such as the ‘Sustainability in Health care by Allocating Resources Effectively’ (SHARE) disinvestment programme in Australia, have also struggled to show impact… |  |
|  | *Lines 386-389 -> indeed. How, though? It would be good to end with your views on how this can be achieved, e.g. solutions to low-value care* | We agree and plan to present recommendations for future de-adoption efforts. However, we do not think this is appropriate in this piece of work. The wider project, of which this is an important piece, will bring together quantitative and qualitative research, patients, clinicians and policymakers views to develop recommendations that will be published in future work. | **Line 460 to 463**  Our project aims to synthesis findings from our quantitative and qualitative work and present these to stakeholders including patients, clinicians, commissioners, and national policy leads with a view to co-producing recommendations to optimise future de-adoption efforts. |  |
|  | *Lines 403-404 -> update as now already beyond that point* | Thanks for spotting. We have removed mention of list 3 as this has recently been removed from the EBI programmes website. | **Line 453** |  |
|  | *Lines 407-410 -> yes! see previous comment onlines 386 onwards. Consider rearranging the discussion a bit to strengthen this narrative and make it more easily visible* | Thank you.  See response above. Our project, as a whole, is working towards evidence-based recommendations with input from patients, policymakers, commissioners, and clinicians. Therefore, we will make more of this point in future publications once we have undertaken more research. | **Line 460 to 463**  Our project aims to synthesis findings from our quantitative and qualitative work and present these to stakeholders including patients, clinicians, commissioners, and national policy leads with a view to co-producing recommendations to optimise future de-adoption efforts. |  |
|  | *Line 418: we need to BETTER understand (I think there is already knowledge on this, but you are right that we need to increase/strengthen it and its application)* | Thanks, we have added ‘better’ to the sentence. | **Line 472**  We need to better understand the enablers and barriers to effective de-adoption to support future de-adoption endeavours. |  |
| **Journal Requirements** | | | | |
| 1. Please ensure that your manuscript meets PLOS ONE's style requirements, including those for file naming. The PLOS ONE style templates can be found at… | | We have edited and are following the style and naming requirements to the best of knowledge. |  | |
| 2. Please state what role the funders took in the study… | | Sorry for not including this. We have added to our funding statements as requested to state that the funders took no role in the study. | **Line 483 to 485**  The funders had no role in study design, data collection and analysis, decision to publish, or preparation of the manuscript | |
| 3. In your Data Availability statement, you have not specified where the minimal data set underlying the results described in your manuscript can be found… | | We have now provided a minimal dataset included in our resubmission.  This includes national counts over time for each of the 17 EBI List 1 procedures and any substitutes used for trend ITS analysis.  Providing counts by age/sex for each CCG (which was used for the SCV variation analysis) would be too disclosive with too many small numbers. Therefore, more detailed data is otherwise available from NHS Digital as outlined in the current Data Availability Statement. | S2 Minimal Underlying Data Set. xslx | |
| 4. We note that Figure 2 in your submission contain map images which may be copyrighted… | | The maps were generated using ArcMap 10.7.1 software. This software is owned by ESRI.  We have added an attribution to the map used in figure 2, as is requested in the link below.  <https://doc.arcgis.com/en/arcgis-online/reference/static-maps.htm>  On this page ESRI state that static maps can be used in  ‘In academic publications (for example, research journals, textbooks, and so on)’. | **Figure 2**  Attribution to attribution to Esri and its licensors has been added to the figure as required. | |
| 5. Please review your reference list to ensure that it is complete and correct. | | References have been reviewed and edited. |  | |
